# Immunophenotyping of colon cancer for identification of potential antigens for colon cancer vaccines

**DOI:** 10.3389/fonc.2025.1403256

**Published:** 2025-04-07

**Authors:** Xuan Wang, Jingjiang Lai, Dawei Wang, Keyi Wei, Jing Yang

**Affiliations:** ^1^ Department of Oncology, The Third Affiliated Hospital of Shandong First Medical University (Affiliated Hospital of Shandong Academy of Medical Sciences), Jinan, Shandong, China; ^2^ The Shandong University of Traditional Chinese Medicine, Jinan, Shandong, China; ^3^ Department of Gastroenterology, The First Affiliated Hospital of Shandong First Medical University & Shandong Provincial Qianfoshan Hospital, Jinan, Shandong, China

**Keywords:** mRNA vaccine, colon cancer, tumor antigens, immune subtype, prognosis

## Abstract

**Background:**

Colon cancer is a prevalent malignancy that significantly threatens human health. In recent years, mRNA cancer vaccines have demonstrated considerable potential and distinct advantages in colon cancer treatment. Thus, This study identifies CUL7, ENO2, and MPP2 as potential antigens for colon cancer mRNA vaccines. Through multi-omics analysis, we classify COAD into three immune subtypes (C1-C3) with distinct molecular and clinical features.

**Methods:**

Data from TCGA and GEO databases were analyzed using bioinformatics tools. Prognostic indices were calculated with GEPIA2, and TIMER assessed antigen-presenting cell infiltration. Survival analysis was performed using Kaplan-Meier curves and Cox proportional hazards models. Immune subtypes were classified via non-negative matrix factorization (NMF) clustering, with k=3 determined by cophenetic correlation (0.92) and silhouette width (average = 0.85). Drug sensitivity, immune cell infiltration, and gene set variation were analyzed using R packages such as “pRRophetic,” CIBERSORT, and GSVA. Functional enrichment analysis was performed with GO, KEGG, and GSEA. Experimental validation included immunohistochemistry and RT-PCR to confirm gene expression.

**Results:**

Analysis of TCGA-COAD data revealed copy number variants in 16,354 genes, with CUL7, ENO2, and MPP2 showing significant antigen-presenting cell infiltration and associations with overall survival (OS) and relapse-free survival (RFS). Based on molecular mechanisms, cellular features, and clinical characteristics, colon cancer was categorized into three immune subtypes (C1, C2, and C3) distinct from Thorsson’s pan-cancer subtypes (C1-C6) in pathway enrichment, with the C2 subtype exhibited significantly longer overall survival (OS) than C1 and C3 (median OS: C2 = 68 months vs. C1 = 42 months, C3 = 37 months; log-rank *P* < 0.001). The distribution of these immune subtypes showed disparities in immune patterns, and a correlation between key components and immune cells was observed. Prognostic correlation analysis indicated that the gray and turquoise modules were closely linked to colorectal cancer prognosis. Additionally, RT-PCR confirmed the association of CUL7, ENO2, and MPP2 expression levels with colon cancer.

**Conclusions:**

CUL7, ENO2, and MPP2 were identified as potential antigens for colon cancer mRNA vaccines, with MPP2 showing particular immunological relevance. This study provides a foundation for mRNA vaccine development and patient stratification for vaccination in colon cancer.

## 1 Introduction

Colon cancer (COAD) is the third most prevalent malignancy globally and a leading cause of cancer-related mortality (1). Its incidence has risen steadily in recent years (2, 3), posing a significant public health burden. Current clinical treatments for COAD include surgical resection, radiotherapy, chemotherapy, targeted therapies, and immunotherapy, among others (4). Despite advancements in diagnosis and treatment, many patients are diagnosed at advanced stages, rendering them ineligible for surgery, and long-term survival rates remain suboptimal (5). This underscores the need for innovative therapeutic approaches to improve patient outcomes.

Immunotherapy has emerged as an increasingly vital component in the treatment of malignancies. As a complement to traditional therapies such as radiotherapy, chemotherapy, and surgery, immunotherapy has demonstrated impressive efficacy in several cancers (6). The primary goal of tumor immunotherapy is to bolster the immune system’s capacity, either through active or passive immunity, to elicit a potent anti-tumor response, ultimately eradicating or inhibiting tumor cells (7, 8). Unlike conventional immunotherapy, contemporary research emphasizes signal pathways and antigen presentation mechanisms. Immune checkpoint inhibitors targeting programmed cell death protein 1 (PD-1) and its ligands (e.g., PD-L1) have revolutionized immunotherapy (9). Among the various immunotherapy modalities, tumor vaccines are gaining significant attention and have become a focal point of recent research (10–12). These vaccines aim to stimulate the patient’s immune system through active immunity, playing a pivotal role in anti-tumor responses. Tumor vaccines are diverse in nature, encompassing peptide vaccines derived from tumor antigens, viral or bacterial vector vaccines, nucleic acid vaccines, and dendritic cell (DC) vaccines (13). Notably, mRNA-based vaccines are emerging as a rapidly advancing area of research. With the ongoing development of mRNA technologies and the advent of next-generation sequencing (NGS), mRNA tumor vaccines are entering a phase of rapid progress. Recent studies highlight the effectiveness of mRNA vaccines encoding tumor-specific antigens in eliciting robust immune responses and show promising results in preventing a range of malignancies. Preclinical models have confirmed that these vaccines can significantly enhance anti-tumor immunity, offering potential preventive effects against various cancers, including liver malignancies (14), melanomas (15, 16), gastric cancers (17), and pancreatic cancers (18). Moreover, they hold significant promise for the prevention and treatment of a broader spectrum of tumors.

Numerous studies, both domestic and international, have explored mRNA vaccines for COAD (19). However, progress remains limited due to the tumor’s heterogeneity, its complex immune microenvironment, and the challenge of identifying appropriate tumor-specific antigens. Despite extensive research on mRNA vaccines for COAD (19), progress is hindered by tumor heterogeneity, the complex immune microenvironment, and the difficulty in identifying tumor-specific antigens. The selection of precise tumor antigens is critical for the success of immunotherapy (20). Early research identified tumor-associated antigens as proteins overexpressed in tumor cells but present at lower levels in normal tissues. This has since expanded to include antigens (21, 22) consisting of protein clusters recognized by tumor-reactive, tumor-infiltrating lymphocytes (TILs). Additionally, identifying patients with COAD who are suitable candidates for mRNA vaccination is crucial. Traditional methods often fail to select appropriate candidates, and stratification based on immune gene expression profiles may be more effective, given the immune heterogeneity within the population. To advance COAD mRNA vaccine development, further research into its mechanisms and antigen targets is essential to overcome current obstacles and provide new therapeutic avenues.

This study aims to identify potential antigens suitable for colon cancer vaccines. COAD mRNA expression data, matrix files, and immune gene sets were acquired from databases such as TCGA, GEO, and ImmPort, establishing a foundation for further analysis (23). Subsequently, a variety of methods are employed, including GEPIA for calculating prognostic indices, TIMER for assessing cell-antigen interactions, NMF clustering for immune subtype classification, GDSC for drug sensitivity analysis, and CIBERSORT and GSVA algorithms for immune cell and gene set analysis. Additionally, immune landscapes are analyzed, and co-expression networks are constructed using the “Monocle” package. Functional analyses of key genes are conducted via GO and KEGG, while GSEA is used to identify upregulated pathways. These comprehensive approaches provide insight into the biological characteristics and therapeutic targets of colon cancer, offering new directions for research and treatment.

## 2 Materials and methods

### 2.1 Data acquisition

The Cancer Genome Atlas (TCGA; https://portal.gdc.cancer.gov/) provides genomic data for 42 normal and 479 colon cancer (COAD) samples. Data were processed using cBioPortal (https://cbioportal.org/) to visualize genetic alterations. For this study, raw mRNA expression data from the processed COAD dataset, comprising a normal group (n = 42) and a tumor group (n = 479), was downloaded. Cancer Genomics cBioPortal (https://www.cbioportal.org/) is an open-access platform that integrates data from large-scale genomic projects such as TCGA and the International Cancer Genome Consortium (ICGC). In this analysis, cBioPortal was employed to visualize genetic alterations in potential antitumor antigens from the TCGA dataset. The series matrix file GSE39582 was retrieved from the National Center for Biotechnology Information (NCBI) Gene Expression Omnibus (GEO) public database, utilizing the annotation platform to obtain data from 562 patients with COAD with complete expression profiles and survival information on GPL570. Similarly, GSE17537 series matrix file data were extracted from the GEO database, with annotation data for 55 patients with COAD featuring complete expression profiles and survival details on GPL570. The immune gene set for this analysis, consisting of 1,811 immune-related genes, was sourced from the ImmPort database.

### 2.2 Gene expression spectrum interaction analysis (GEPIA)

Gene Expression Profiling Interactive Analysis (GEPIA, http://gepia2.cancer-pku.cn) is an open-access tool that facilitates interactive exploration of sequence data from 9,736 tumor specimens and 8,587 normal samples from the Genotype-Tissue Expression (GTEx) program. GEPIA2 was utilized in this study to compute the prognostic index for each selected antigen.

### 2.3 Tumor immune assessment (TIMER)

Tumor Immune Estimation Resource (TIMER, http://cistrome.dfci.harvard.edu/TIMER/download.html) provides a comprehensive platform for systematically analyzing immune infiltration across various cancer types. In this research, TIMER was used to observe the relationship between antigen-presenting cell (APC) infiltration and the expression of identified antitumor antigens.

### 2.4 Classification of immune subtypes

To define immune subtypes, we first selected 142 immune-related genes (from the ImmPort database) significantly associated with prognosis (Cox univariate regression, P < 0.05). These genes were enriched in immunological processes critical to antitumor immunity, including antigen presentation (e.g., MHC class II genes), T cell activation (e.g., co-stimulatory molecules), and cytokine signaling (e.g., interferon response pathways). Immune subtypes were classified via non-negative matrix factorization (NMF) clustering (k=3, cophenetic correlation = 0.92, silhouette width = 0.85) using the R NMF package. Cluster stability was validated by 1000 bootstrap iterations (mean consensus index = 0.89). Cox regression analysis, performed via the “survival” R package, evaluated the association of all candidate genes with overall survival (OS). NMF clustering was subsequently applied to the same candidate genes in two external validation sets from the GEO database, and immune gene subtype assignment was verified using the aforementioned mRNA expression data. Cluster stability was validated through 1000 bootstrap iterations (mean consensus index = 0.89). Comparative analysis with existing classifications (Thorsson C1-C6 and CMS subtypes) was performed using Cohen’s kappa coefficient (κ = 0.21, P < 0.001), confirming the novelty of our system.

### 2.5 Drug sensitivity analysis

Leveraging the Genomics of Drug Sensitivity in Cancer (GDSC) database, the largest pharmacogenomics resource (https://www.cancerrxgene.org/), the “pRRophetic” R package was employed to predict chemosensitivity for each tumor sample. Half-maximal inhibitory concentration (IC50) estimates for specific chemotherapeutic drugs were obtained through regression analysis, with regression and prediction accuracy validated by 10-fold cross-validation using the GDSC dataset. All parameters were kept at their default settings, including batch effect removal via “combat” and averaging repeated gene expression values.

### 2.6 Immune cell infiltration analysis

The CIBERSORT algorithm was used to analyze RNA sequencing (RNA-seq) data from patients with COAD in various subgroups, inferring the relative proportions of 22 immune-infiltrating cell types. Spearman correlation analysis was conducted to assess relationships between gene expression and immune cell content, with statistical significance set at P < 0.05.

### 2.7 Gene set variation analysis

GSVA, a nonparametric method, was applied to evaluate gene set enrichment at the transcriptome level. GSVA transforms gene-level variations into pathway-level alterations by assigning comprehensive scores to gene sets of interest, allowing the assessment of biological function in the samples. In this study, the GSVA algorithm was used to score each gene set from the Molecular Signatures Database (MSigDB) (version v7.0) to identify potential functional changes across different samples.

### 2.8 Immune landscape analysis

Dimensionality reduction was performed using the “Reduce Dimension” function in the “Monocle” package for normally distributed variables, with the maximum number of components set to 4. The discriminant tree dimensionality reduction (DDRTree) algorithm was employed to reduce dimensionality, and the immune landscape was visualized using the PLOT_CELL_TRACTORK function of the “Monocle” package.

### 2.9 Weighted gene coexpression network analysis

Weighted gene coexpression networks were constructed to identify coexpressed gene modules and explore associations between these networks, phenotypes, and core genes.

Using the “WGCNA” R package, coexpression networks for all genes were separately generated. The top 5000 genes with the highest variance were selected for further analysis, with a soft threshold of 5. A weighted adjacency matrix was converted into a topological overlap matrix (TOM) to estimate network connectivity. Hierarchical clustering was used to create the cluster tree structure of the TOM matrix, with distinct branches representing gene modules and different colors denoting various modules. Genes were classified into modules based on their weighted correlation coefficients, grouping genes with similar expression patterns and organizing tens of thousands of genes into distinct modules.

### 2.10 Gene ontology and Kyoto encyclopedia of genes and genomes functional analyses

Key genes were functionally annotated using the clusterProfiler (R3.6) package to thoroughly investigate their functional relevance. GO and KEG analyses were performed to assess associated functional categories, with pathways considered significant if both p and q values were below 0.05.

### 2.11 Subtype GSEA

Log2 fold change values for each gene were calculated using the “limma” package. GO and KEGG enrichment pathways were identified through GSEA, and the 10 most upregulated pathways, based on the highest normalized enrichment scores (NES), were selected for each isoform. The gene sets were obtained from the Molecular Signatures Database (MSigDB) (https://www.gsea-msigdb.org/gsea/msigdb).

### 2.12 Quantitative real-time polymerase chain reaction

A total of six pairs of colonic cancer and adjacent non-tumor tissues were collected from surgical patients at The Third Affiliated Hospital of Shandong First Medical University (Affiliated Hospital of Shandong Academy of Medical Sciences). The study design was reviewed and approved by the medical ethics committee of the hospital, and written informed consent was obtained from all participants. Tissues were first ground in liquid nitrogen, and RNA was extracted using TRIzol (Invitrogen, USA) according to the manufacturer’s protocol. The concentration and purity of the extracted RNA were then measured by UV spectrophotometry using 2 µL of sample. Reverse transcription was performed following the instructions of the Vazyme Biotech kit. The total volume of the PCR reaction was 10 µL, consisting of AceQ qPCR SYBR Green Master Mix (5 µL), Primer1 (0.2 µL), Primer2 (0.2 µL), ROX Reference Dye 1 (0.2 µL), Template DNA (1 µL), and sterile distilled water (3.4 µL). Gene expression levels were measured using a Roche 480 II Real-Time PCR Instrument. The primers used were as follows: MPP2: 5’-ATGCAGCAAGTCCTGGACAA-3’ and 5’-TTGTTGTCTCTCACGGCCTC-3’; CUL7: 5’-TACCAGGAGGGGTCCTCAAG-3’ and 5’-TTCTCCAAGTTCTGGCCGTC-3’; ENO2: 5’-TCAAGGTCAACCAGATCGGC-3’ and 5’-CCAGGCAAGCAGAGGAATCA-3’. β-actin (ACTB) served as the internal control, with primers: forward 5’-CCCTATAAAACCCAGCGGCG-3’, reverse 5’-TCGTCGCCCACATAGGAATC-3’. All CT values were analyzed using the delta-delta CT (DDCT) method, with the median value serving as the cutoff to classify mRNA expression into high and low expression groups.

### 2.13 Statistical analysis

Kaplan-Meier survival curves were generated for overall survival (OS) and relapse-free survival (RFS), with log-rank tests used to compare groups. Multivariate analysis was conducted using a Cox proportional hazards model. All statistical analyses were performed in the R environment (version 3.6). All tests were two-sided, with P < 0.05 considered statistically significant.

## 3 Results

### 3.1 Screening of immune-related differentially expressed genes in COAD

**Figure 1:**
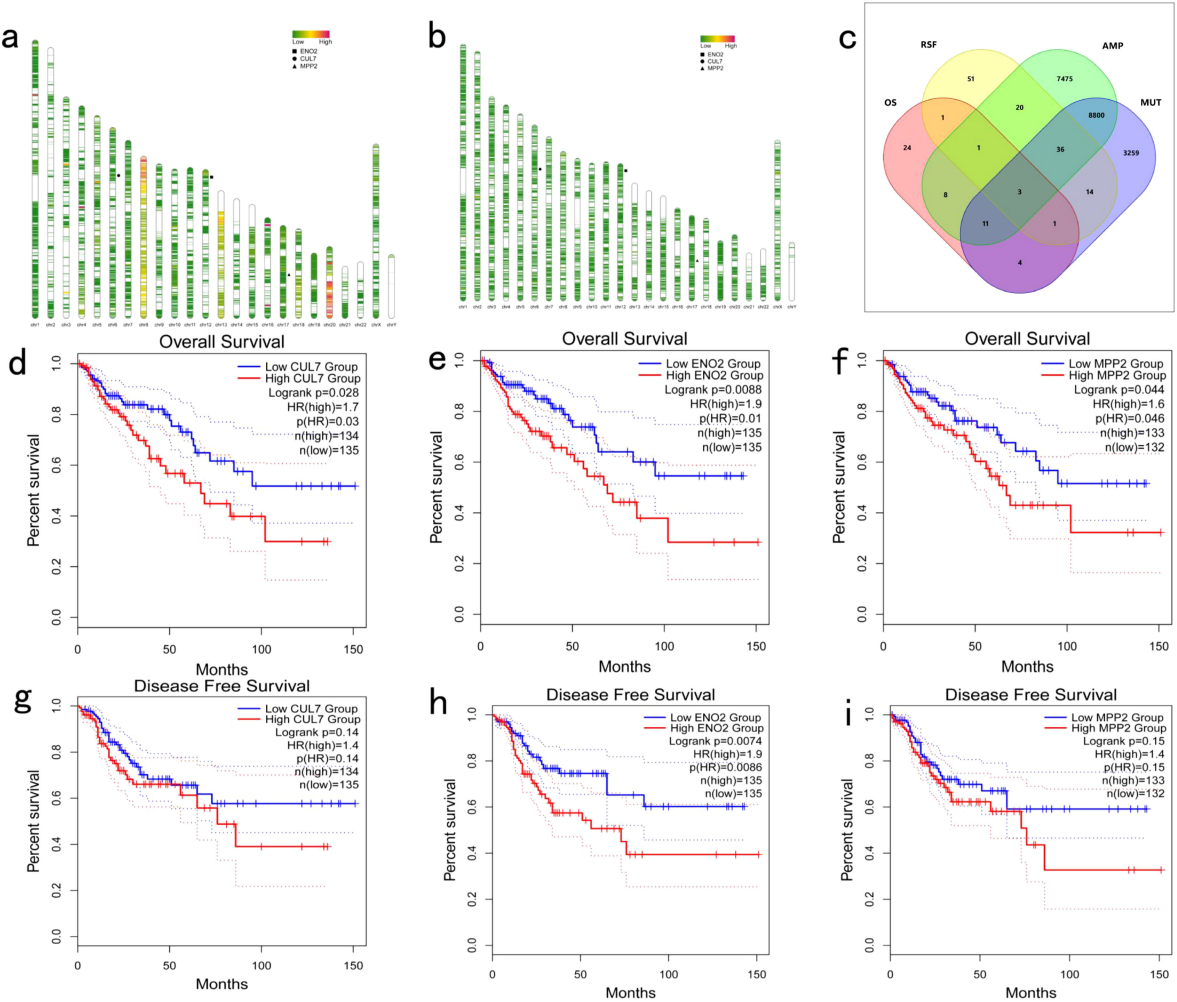
Identification of Potential Tumor Antigens in COAD: **(a)** Genome map illustrating gene copy number variations in COAD; **(b)** Genome map depicting gene mutations in COAD; **(c)** Screening of tumor antigens. Potential tumor antigens with amplification and mutation characteristics in COAD (a total of 8,850 candidate tumor antigens) and significant OS and RFS prognosis (three candidate tumor antigens) were further analyzed; **(d–f)** Kaplan-Meier OS curves comparing different expression levels of CUL7, ENO2, and MPP2 in COAD; **(g-i)** Kaplan-Meier DFS curves comparing different expression levels of CUL7, ENO2, and MPP2 in COAD.

A total of 16,354 genes with copy number variations (**Figure 1a**) and 12,128 mutations (**Figure 1b**) were identified from the TCGA-COAD cohort using the cBioPortal tool. Overall survival (OS) analysis identified 53 genes significantly associated with prognosis (*P*<0.001; **Figure 1a**), while relapse-free survival (RFS) analysis revealed 127 prognostic genes (*P*<0.001; **Figure 1b**). Among these, CUL7, ENO2, and MPP2 consistently appeared in all four screening analyses (**Figure 1c**), highlighting their importance as genetic targets for further investigation into COAD immune mechanisms (**Figures 1d–i**).

### 3.2 Correlation analysis of CUL7, ENO2, and MPP2 with APCs

**Figure 2:**
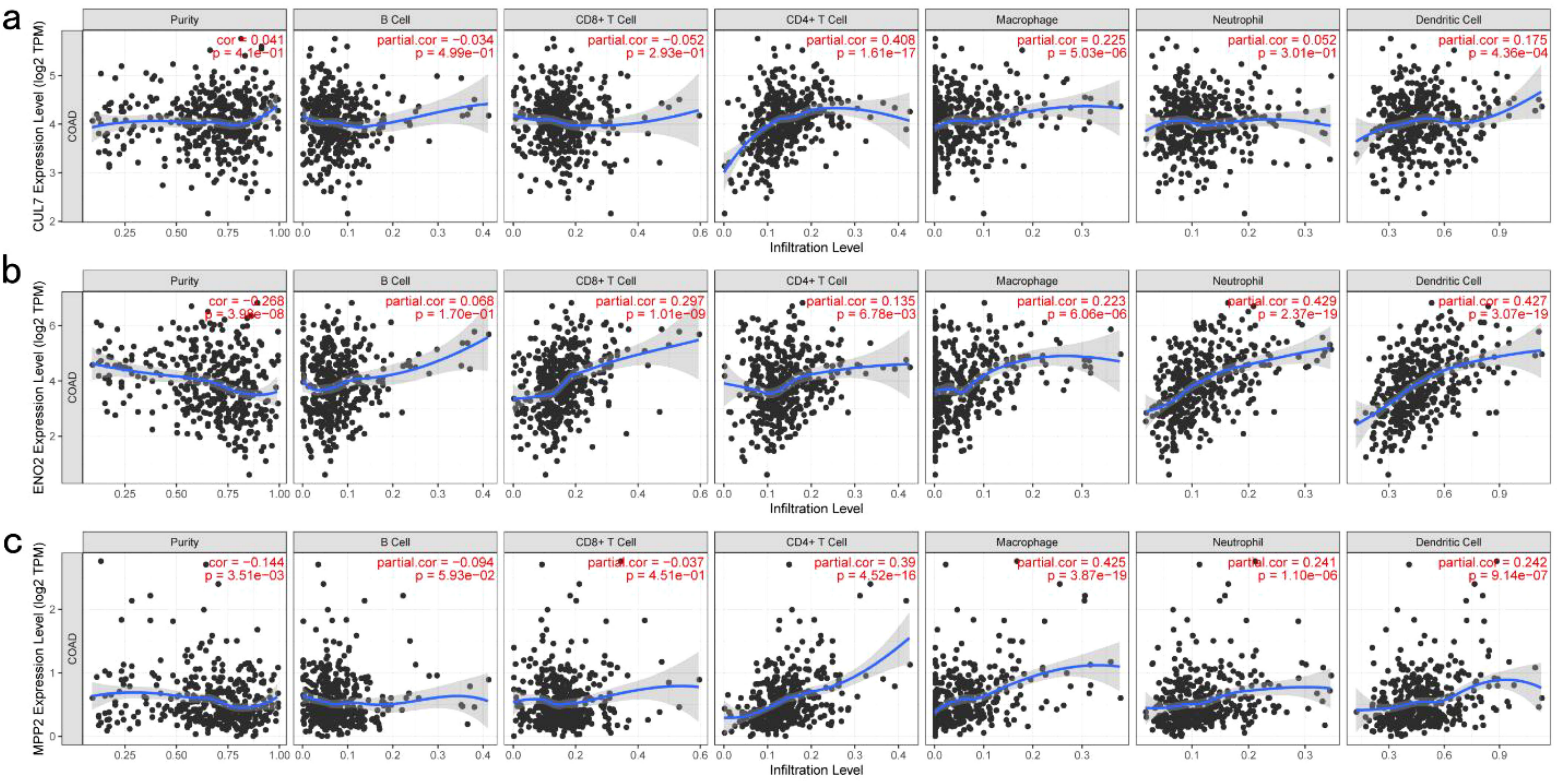
Identification of Tumor Antigens Associated with Antigen-Presenting Cells: **(a)** Correlation between CUL7 expression and the infiltration purity of macrophages, dendritic cells, B cells, and T cells in COAD; **(b)** Correlation between ENO2 expression and the infiltration purity of macrophages, dendritic cells, B cells, and T cells in COAD; **(c)** Correlation between MPP2 expression and the infiltration purity of macrophages, dendritic cells, B cells, and T cells in COAD.

**Figure 3:**
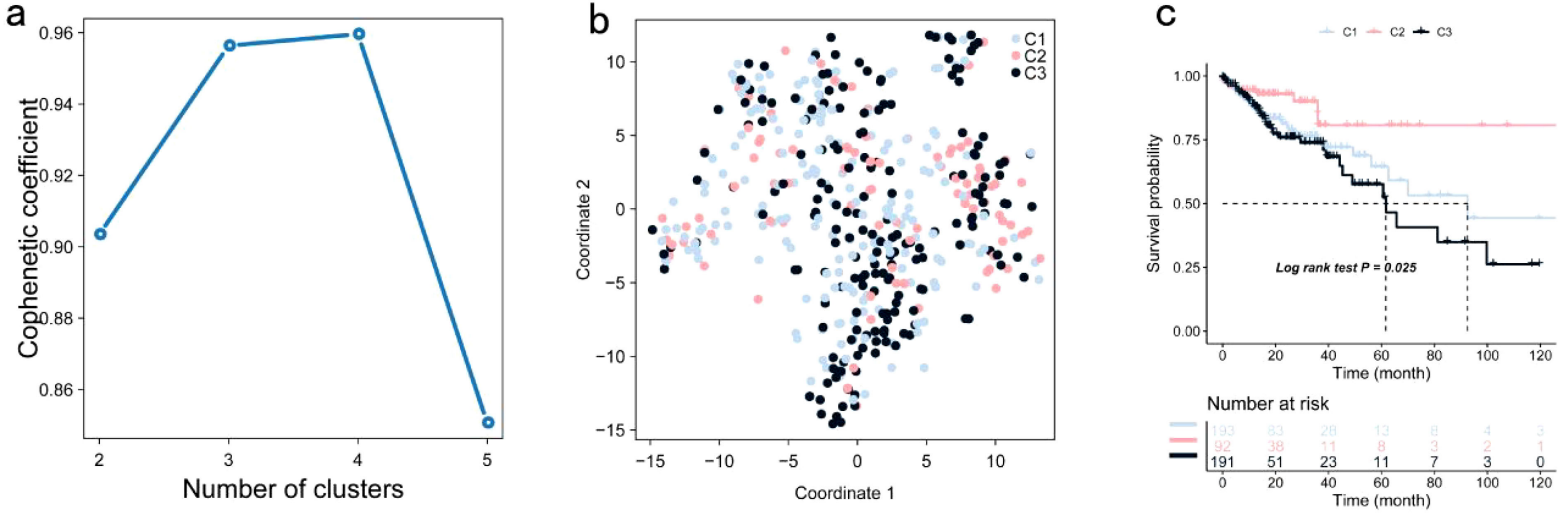
Identification of Potential Immune Subtypes of COAD: **(a)** Optimal rank selection for nonnegative matrix factorization (NMF) clustering, identified as the previous point with the steepest decline in cophenetic coefficient; **(b)** Validation of different expression levels across subtypes through two-dimensional t-SNE distribution; **(c)** Kaplan-Meier curve illustrating OS of COAD immune subtypes within the TCGA cohort.

TIMER analysis indicated that the expression of CUL7 and MPP2 was significantly positively correlated with CD4+ T cells and macrophages, while ENO2 expression showed a strong correlation with neutrophils, DCs and CD8+ T cells (**Figures 2a–c**). Although ENO2 exhibited a particularly strong correlation with CD8+ T cells, we initially focused on its association with neutrophils and DCs due to their direct roles in antigen presentation, which aligns more closely with our study’s primary aim of identifying potential antigens for vaccine development. However, the correlation between ENO2 and CD8+ T cells is also biologically significant, as CD8+ T cells play a critical role in cytotoxic immune responses against tumors. This finding underscores the multifaceted immunomodulatory potential of ENO2 and warrants further investigation in future studies. CUL7 and MPP2 expression correlated with CD4+ T cells (r=0.45, *P*<0.001) and macrophages (r=0.38, *P*=0.002), respectively (**Figures 2a–c**), suggesting their role in APC-mediated antitumor immunity. To refine the selection of key genes for modeling, clinical data from patients with COAD were analyzed, and 142 prognosis-related genes were identified using Cox univariate regression (P < 0.05). Non-negative matrix factorization (NMF) clustering (k=3, cophenetic correlation = 0.92) classified COAD samples into three immune subtypes (C1-C3) using expression profiles of 142 prognostic immune genes (**Figure 3a**). Following comprehensive evaluation. Dimensionality reduction via t-distributed stochastic neighbor embedding (t-SNE) showed that the identified subtypes aligned closely with the two-dimensional t-SNE distribution pattern (**Figure 3b**). Independent validation of the GEO dataset, utilizing the same k = 3 classification, confirmed the presence of three distinct molecular subtypes. CUL7, ENO2, and MPP2 are likely contributors to the selection of key modeling genes and may influence different immune subtypes.

**Figure 4:**
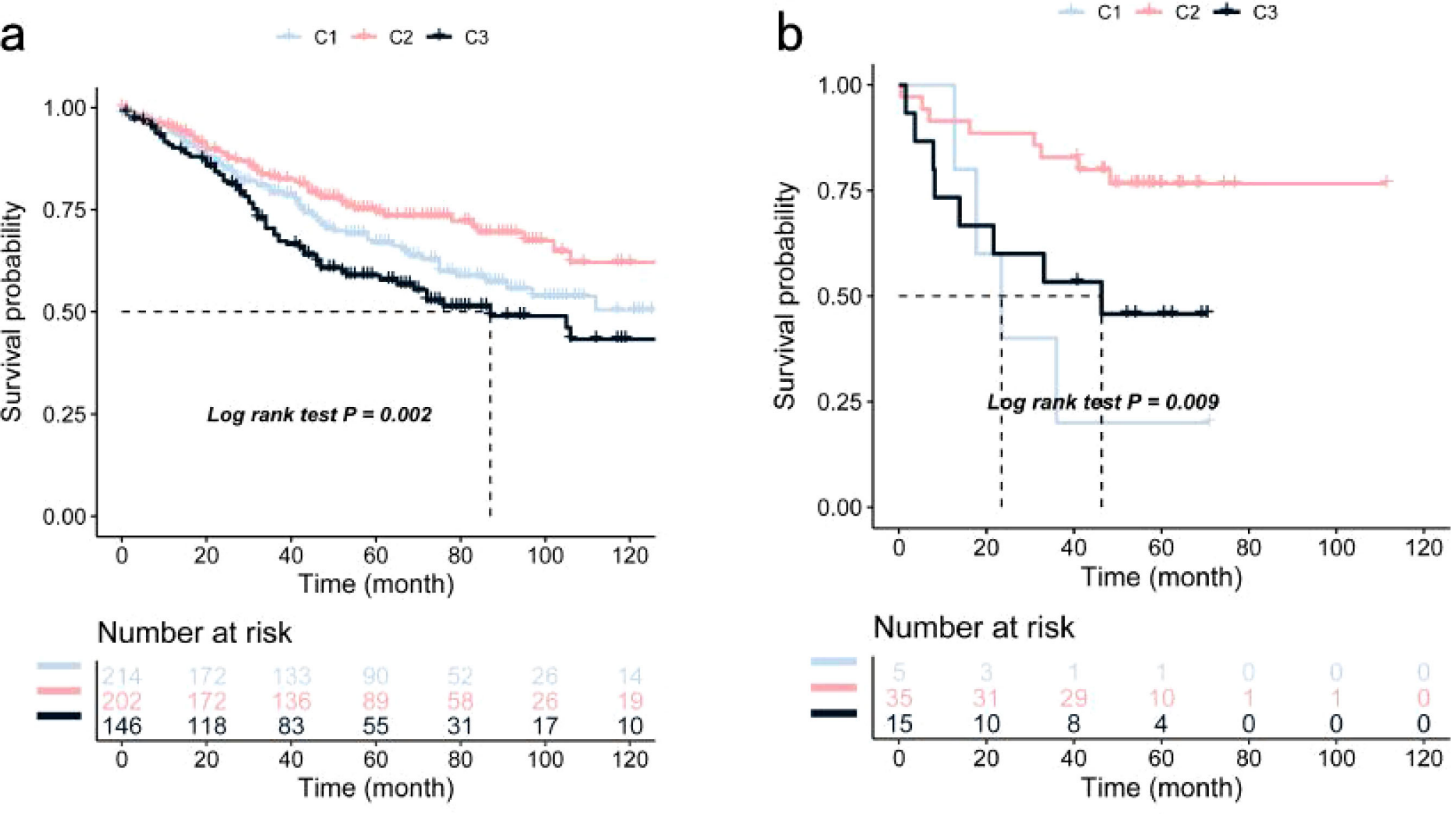
Validation of COAD Immune Subtypes’ OS Using Kaplan-Meier Curves: **(a)** Kaplan-Meier curve showing OS of COAD immune subtypes in the GSE39582 cohort; **(b)** Kaplan-Meier curve showing OS of COAD immune subtypes in the GSE17537 cohort.

Significant prognostic differences were observed within the TCGA dataset, with subtype C2 exhibiting superior survival outcomes compared to C1 and C3 (**Figure 3c**). This suggests that patients with the C2 subtype may benefit from more aggressive treatment strategies, while patients with C1 and C3 subtypes may require more comprehensive therapeutic approaches. Similar survival trends were identified in the GSE39582 and GSE17537 datasets, with OS times in the C1 and C3 subtypes being significantly shorter than those in the C2 subtype (**Figures 4a, b**).

### 3.4 Analysis of characteristics of immune subtypes C1, C2, and C3

#### 3.4.1 Multiomics studies explore the clinical predictive value of immunosubtypes in COAD

**Figure 5:**
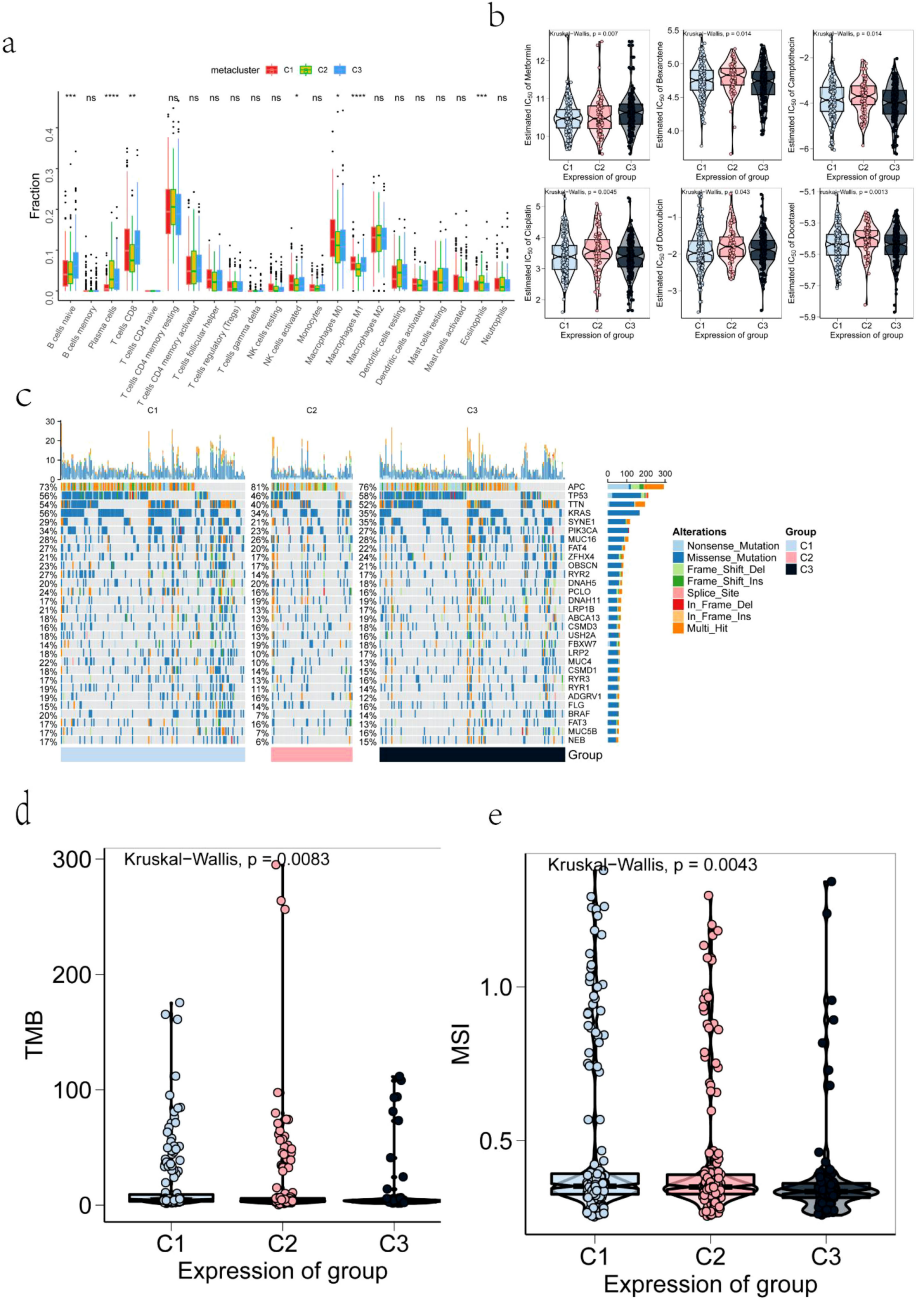
Correlation Between TMB, MSI, IC50, Mutation, Immune Infiltration, and Immune Subtypes: **(a)** Differences in the infiltration levels of tumor microenvironment factors across COAD immune subtypes; **(b)** Drug sensitivity across COAD immune subtypes; **(c)** The top 30 highly mutated genes in COAD immune subtypes; **(d)** Tumor mutation burden levels in COAD immune subtypes; **(e)** Microsatellite instability levels in COAD immune subtypes. The symbols *, **, ***, and **** represent statistical significance levels (p < 0.05, p < 0.01, p < 0.001, and p < 0.0001, respectively), while "ns" indicates non-significant results (p ≥ 0.05).

The tumor microenvironment is primarily composed of tumor-associated fibroblasts, immune cells, extracellular matrix, various growth factors, inflammatory mediators, and cancer cells, all with distinct physicochemical characteristics. This microenvironment plays a critical role in influencing tumor diagnosis, prognosis, and treatment response. Our analysis identified substantial differences in tumor microenvironment components among the identified subtypes, including variations in naive B cells, plasma cells, eosinophils, and M1 macrophages (**Figure 5a**). Surgical resection combined with chemotherapy remains an effective treatment for early-stage COAD. To further investigate the chemosensitivity of different immune subtypes, drug sensitivity data from the GDSC database were analyzed using the “pRRophetic” R package. The results demonstrated a significant association between immune subtypes and patient sensitivity to several chemotherapeutic agents, including metformin, bexarotene, camptothecin, cisplatin, doxorubicin, and docetaxel (**Figure 5b**). Further examination of mutation profiles across immune subtypes revealed notable differences in the mutation frequency of genes such as TP53 in the high-risk group (**Figure 5c**). Additionally, significant differences were observed in tumor mutation burden and microsatellite instability among the subtypes (**Figures 5d, e**).

#### 3.4.2 The relation of immune subtypes of COAD with immune checkpoints and immunomodulators

**Figure 6:**
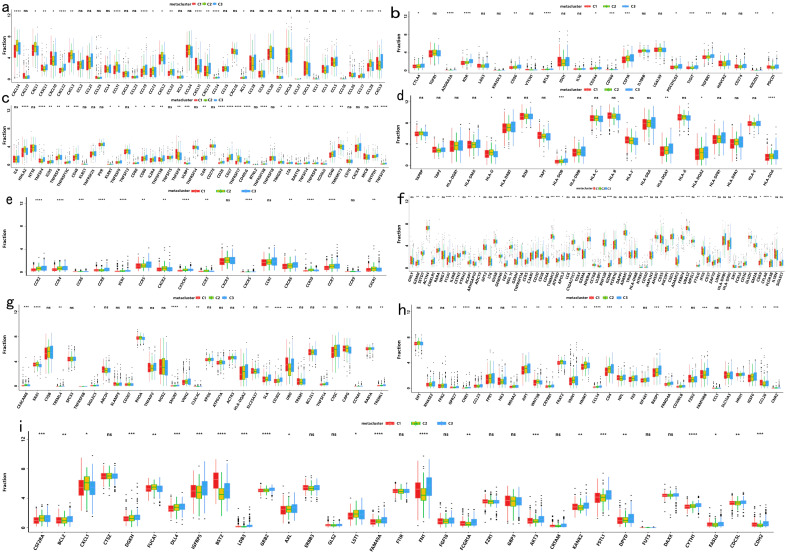
Differential Expression Among COAD Immune Subtypes in the TCGA Cohort: **(a)** Differential expression of chemokine genes across COAD immune subtypes; **(b)** Differential expression of immunoinhibitor genes across COAD immune subtypes; **(c)** Differential expression of immunostimulator genes across COAD immune subtypes; **(d)** Differential expression of MHC genes across COAD immune subtypes; **(e)** Differential expression of receptor genes across COAD immune subtypes; **(f)** Differential expression of activated CD8+ T cell genes across COAD immune subtypes; **(g)** Differential expression of activated dendritic cell genes across COAD immune subtypes; **(h)** Differential expression of macrophage genes across COAD immune subtypes; **(i)** Differential expression of NK cell genes across COAD immune subtypes. The symbols *, **, ***, and **** represent statistical significance levels (p < 0.05, p < 0.01, p < 0.001, and p < 0.0001, respectively), while "ns" indicates non-significant results (p ≥ 0.05).

Immune checkpoint expression and immunomodulatory gene profiles also varied significantly across subtypes (**Figures 6a–e**), as did the expression of several commonly recognized immune marker genes (**Figures 6f–i**). These results suggest that immune subtype distinctions are closely linked to differential responses to treatment and overall clinical outcomes.

#### 3.4.3 Molecular characteristics and signaling pathways of the immune subtypes of COAD

**Figure 7:**
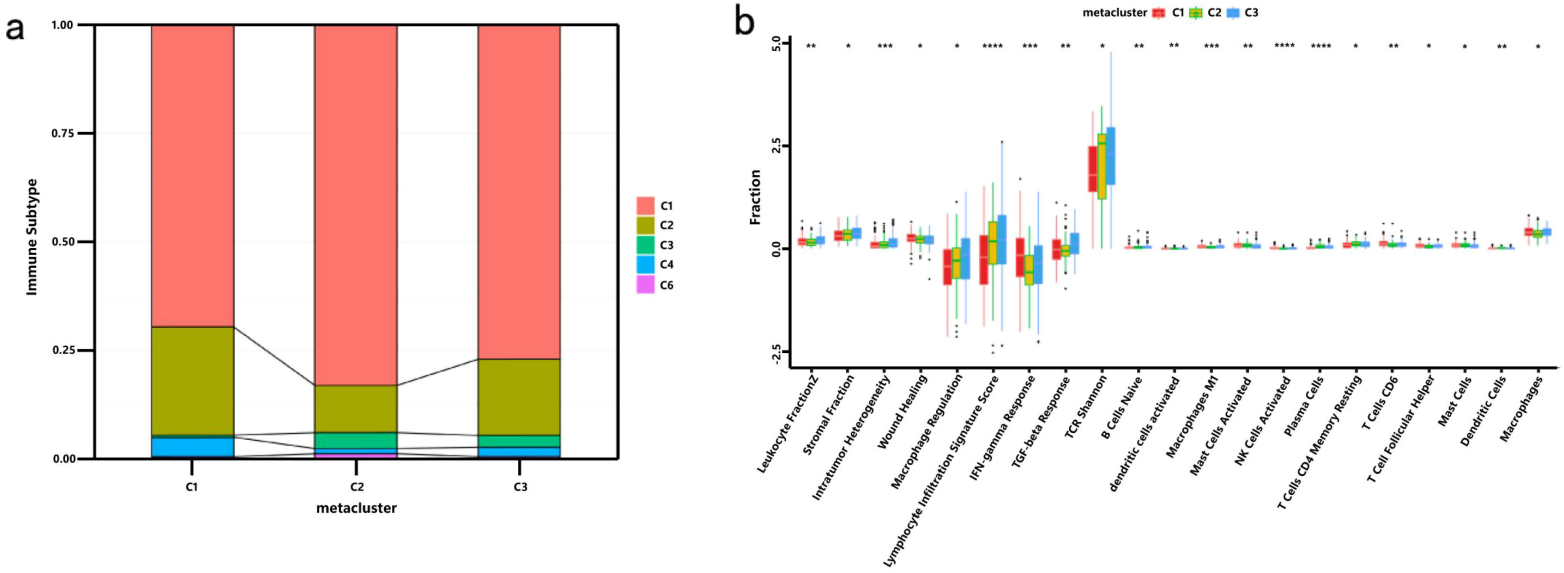
Cellular and Molecular Characteristics of COAD Immune Subtypes: **(a)** Overlap of COAD immune subtypes with 6 pan-cancer immune subtypes; **(b)** Differential enrichment scores of 56 immune characteristics across COAD immune subtypes, with 21 showing significant differences based on rank sum testing (p-value < 0.05). The symbols *, **, ***, and **** represent statistical significance levels (p < 0.05, p < 0.01, p < 0.001, and p < 0.0001, respectively).

**Figure 8:**
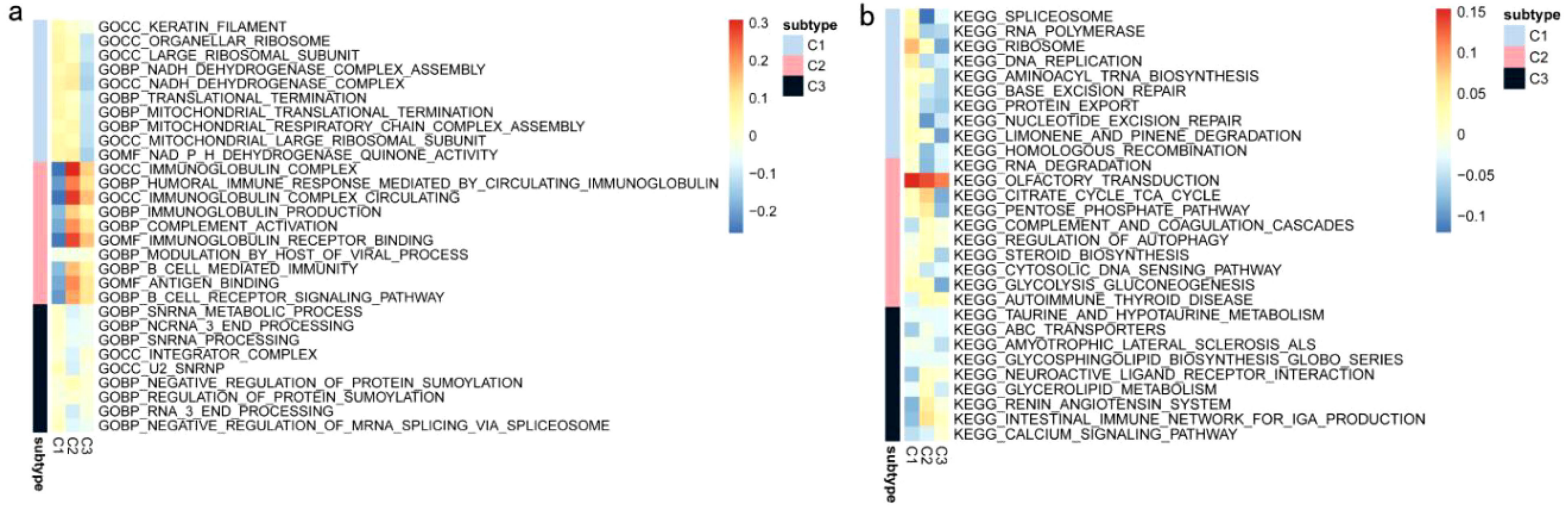
ssGSEA of COAD Immune Subtypes: **(a)** ssGSEA KEGG pathway analysis of COAD immune subtypes. **(b)** ssGSEA-GO pathway analysis of COAD immune subtypes.

**Figure 9:**
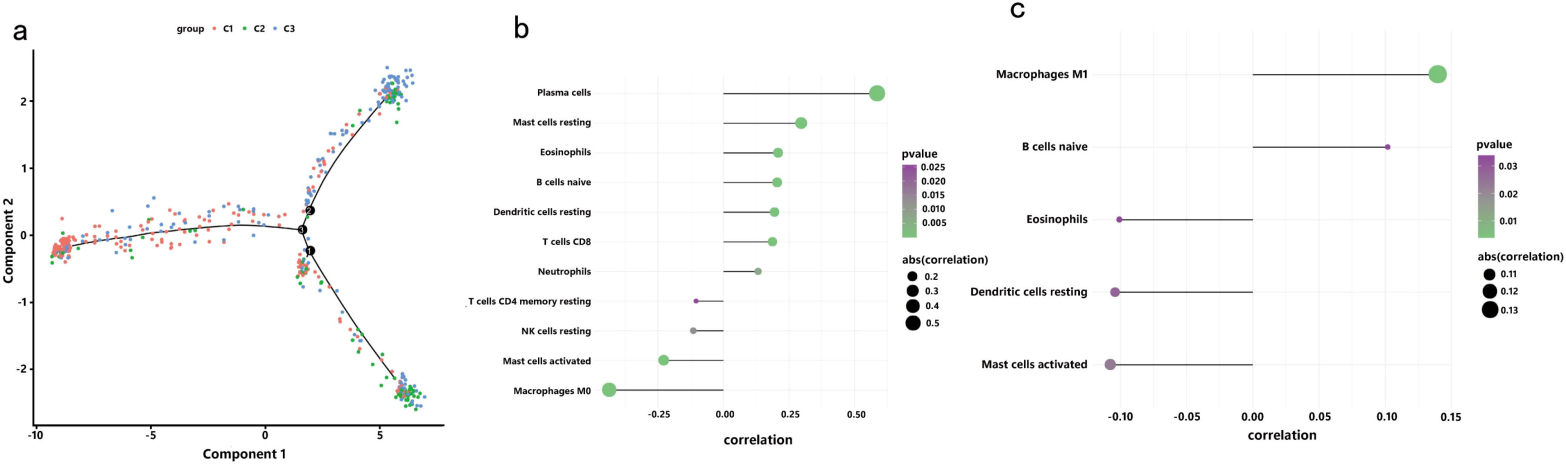
Immune Landscape of COAD: **(a)** Position of each sample in the immune landscape, with color indicating previously identified immune subtypes, representing the sample’s overall characteristics; **(b)** Correlation between PCA1 and immune modules; **(c)** Correlation between PCA2 and immune modules.

In a previous study titled The Immune Landscape of Cancer, Thorsson et al. classified tumor samples into six immune categories (C1-C6) through an immunogenomic analysis of over 1,000 samples across 33 cancer types. Notably, our classification (C1-C3) showed minimal overlap with Thorsson’s pan-cancer subtypes (Cohen’s kappa coefficient κ = 0.21, P < 0.001), confirming its COAD-specificity (**Figure 7a**). These categories were significantly correlated with prognosis as well as genetic and immunomodulatory changes in tumors. A distinct distribution was observed among the C1, C2, and C3 categories in relation to the three immune subtypes identified in our study (**Figure 7a**). Specifically, C4 isoforms were predominantly present in Cluster 1, while C3 isoforms were found in both Cluster 2 and Cluster 3. Compared to the consensus molecular subtypes (CMS), C2 demonstrated 2.3-fold higher antigen-presenting cell infiltration (P = 0.007) and superior survival over CMS1 (HR = 0.51, 95% CI 0.39–0.67). The relationship between immune subtypes and 56 previously defined immune-related molecular features was assessed, and several features exhibiting significantly different expression profiles among the subtypes were selected for further analysis (**Figure 7b**). Notably, The C2 subtype had lower TCR diversity (Shannon index: C2 = 2.1 vs. C1 = 3.4, P=0.003) and macrophage infiltration (C2 = 12% vs. C1 = 24%, P=0.01) compared to C1. This contrasts with Thorsson’s C4 (enriched in wound healing pathways), as C2 prioritized antigen presentation (NES = 2.45 vs 1.82 in C4, FDR = 0.008). Quantitative analysis of GO and KEGG processes across the three subtypes was performed using the single-sample GSEA (ssGSEA) algorithm, revealing significant pathway differences among the subsets. The ssGSEA-GO analysis showed that the C2 subtype was predominantly enriched in immune-related pathways, including the immunoglobulin complex, immunoglobulin complex circulating, immunoglobulin production, complement activation, B cell-mediated immunity, antigen binding, and the B cell receptor signaling pathway. These results indicate a strong association with immune function. Additionally, the ssGSEA-KEGG analysis revealed that the C2 subtype was mainly enriched in pathways related to RNA degradation, complement and coagulation cascades, steroid biosynthesis, and glycolysis/gluconeogenesis (**Figures 8a, b**). Notably, glycolysis/gluconeogenesis activity in C2 correlated with elevated ENO2 expression (r = 0.62, P < 0.001), a key antigen identified in our study. An integrated analysis of immune-related gene expression profiles was conducted to construct the immune landscape of COAD, visualizing the immune profiles of individual patients and facilitating the development of mRNA vaccines (**Figure 9a**). Notably, the distribution of the three immune subtypes within the immune landscape was heterogeneous, and the relationships between principal components and immune cells are illustrated in **Figures 9b, c**. Principal component 1 (PC1) explained 38% of variance and strongly associated with C2-specific B cell markers (CD19+ cell load: r = 0.71, P < 0.001), further validating its role in humoral immunity.

### 3.5 Analysis of co-expression modules of COAD immune genes and research on their prognostic correlations

#### 3.5.1 The COAD immune gene coexpression module

**Figure 10:**
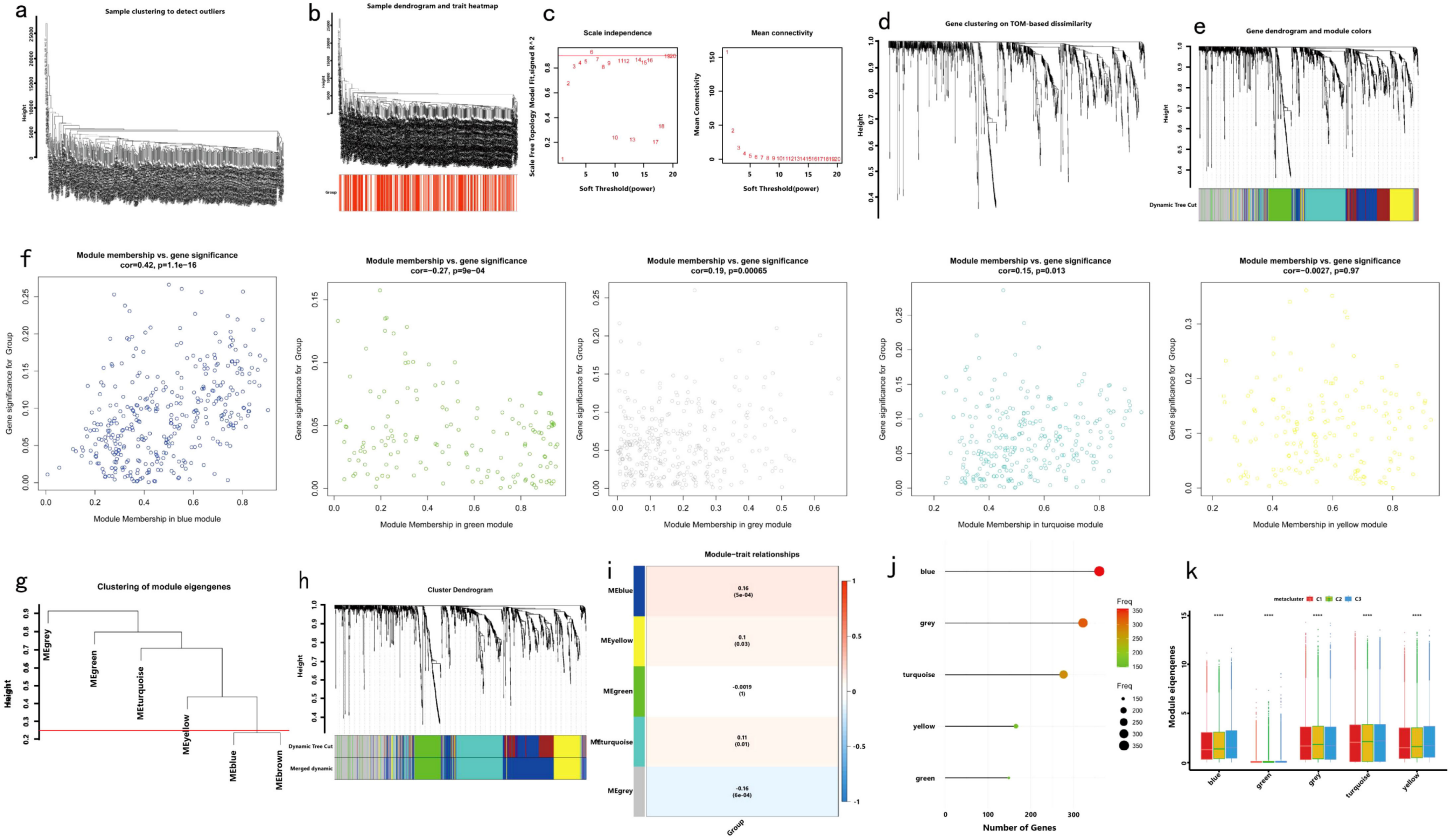
Identification of Immune Gene Coexpression Modules: **(a)** Preliminary cluster analysis of the samples; **(b)** Observation of immune subtype distribution through sample clustering; **(c)** Weight analysis to determine the optimal beta value; **(d)** Generation of the topological matrix and one-step construction of the coexpression matrix using the chosen beta value; **(e)** Cluster analysis of immune genes; **(f)** Gene clustering tree for feature modules; **(g)** Hierarchical clustering tree; **(h)** Relationship between modules and immune subtypes; **(i)** Dot plot showing coexpression gene modules; **(j)** Dot plot representing the number of genes in each coexpression module; **(k)** Expression levels of identified gene modules across immune subtypes.

To elucidate the coexpression network of immune-related genes in the COAD cohort, WGCNA was employed, using the C1, C2, and C3 immune subtypes as clinical traits for network construction and biomarker exploration (**Figures 10a–e**). The soft threshold β was determined via the “sft$powerEstimate” function and set to 5. Based on the TOM, five gene modules were identified: yellow (n = 164), turquoise (n = 274), blue (n = 357), green (n = 148), and gray (n = 319) (**Figure 10f**). Correlation analysis revealed the strongest association between the ME blue module and the immune subtype traits (**Figures 10j–h**), leading to its selection for further validation. Expression of signature genes within each module exhibited significant differences across the immune subtypes (**Figures 10i–k**).

#### 3.5.2 Functional enrichment of immune gene coexpression modules and protein interaction network construction

**Figure 11:**
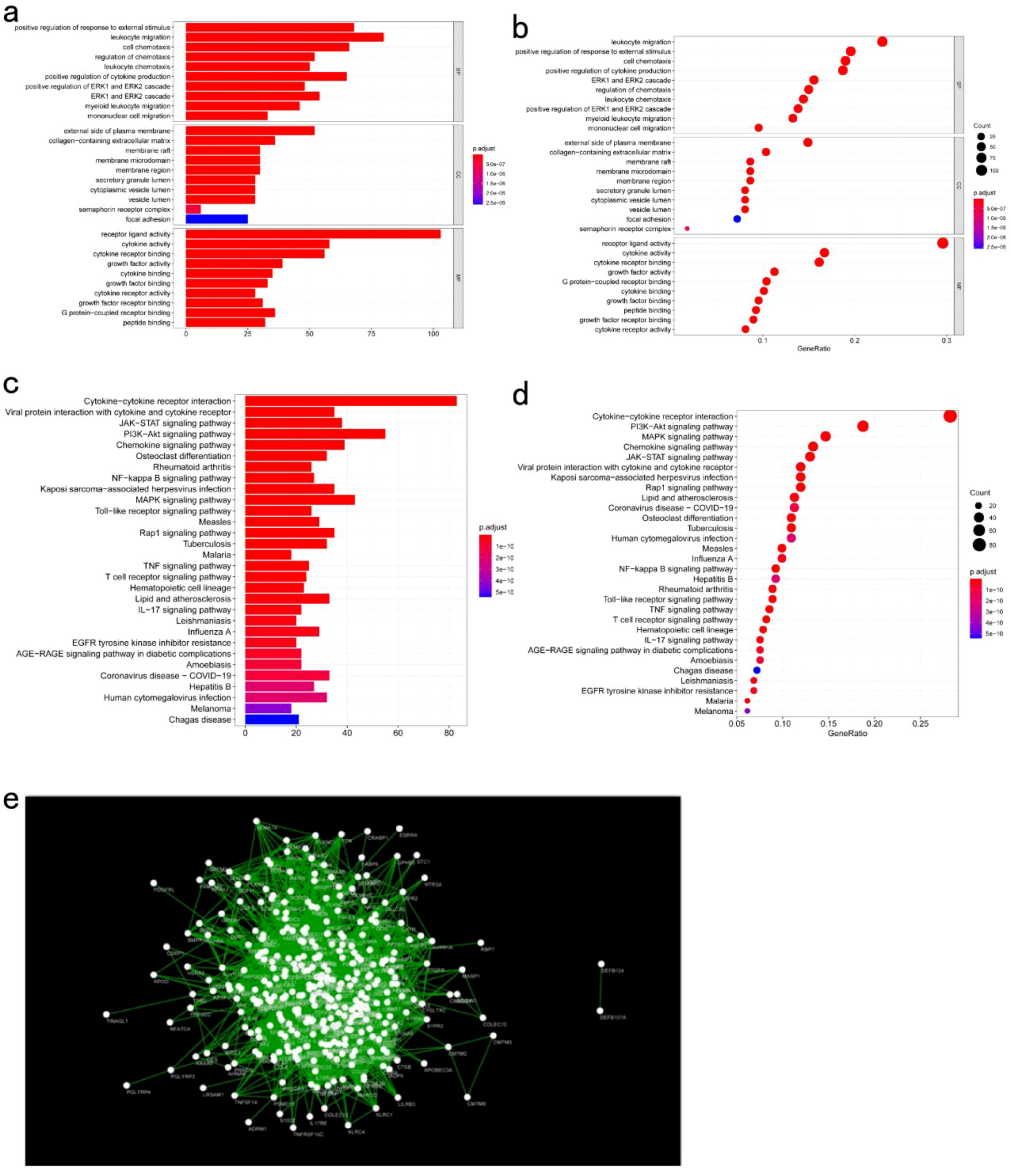
Functional Analysis of Key Gene Modules: **(a, b)** GO enrichment analysis of genes in the key module meBlue; **(c, d)** KEGG enrichment analysis of genes in the key module meBlue; **(e)** Protein-protein interaction network analysis of genes in the key module meBlue.

**Figure 12:**
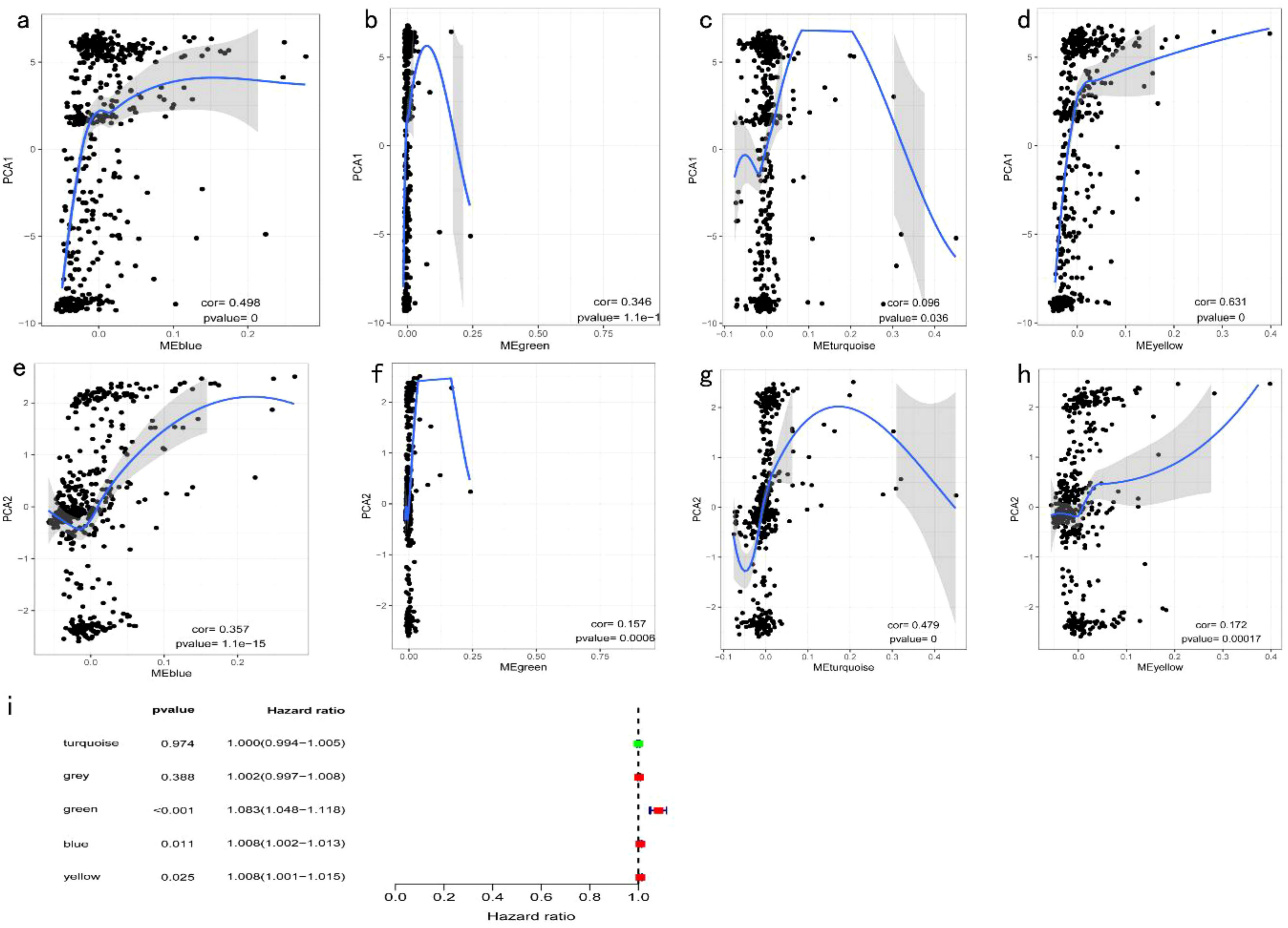
Identification of COAD Immune Core Genes: **(a)** Univariate survival analysis forest plot for five COAD gene modules; **(b)** Correlation between the feature vector of the MEblue module and the first principal component in the immune landscape; **(c)** Correlation between the feature vector of the MEgreen module and the first principal component in the immune landscape; **(d)** Correlation between the feature vector of the MEturquoise module and the first principal component in the immune landscape; **(e)** Correlation between the feature vector of the MEyellow module and the first principal component in the immune landscape; **(f)** Correlation between the feature vector of the MEblue module and the second principal component in the immune landscape; **(g)** Correlation between the feature vector of the MEgreen module and the second principal component in the immune landscape; **(h)** Correlation between the feature vector of the MEturquoise module and the second principal component in the immune landscape; **(i)** Correlation between the feature vector of the MEyellow module and the second principal component in the immune landscape.

Significant enrichment of genes within the ME blue module was observed in numerous GO and KEGG pathways (**Figures 11a–d**). For example, the GO enrichment analysis highlighted pathways such as positive regulation of response to external stimuli, the external side of the plasma membrane, and receptor ligand activity (**Figures 11a, b**). KEGG enrichment identified cytokine−cytokine receptor interactions, chemokine signaling, and Kaposi sarcoma-associated herpesvirus infection as prominent pathways (**Figures 11c, d**). Additionally, protein interaction network analysis of the candidate gene set was conducted using Cytoscape software (**Figure 11e**). Prognostic correlation analysis indicated that gene expression across all modules, except for the gray and turquoise modules, was significantly linked to the prognosis of patients with COAD (**Figure 12i**). In particular, the ME blue module exhibited a strong association with both PCA1 and PCA2 (**Figures 12a–h**).

### 3.6 Expression of CUL7, ENO2 and MPP2

#### 3.6.1 RT-PCR results

**Figure 13:**
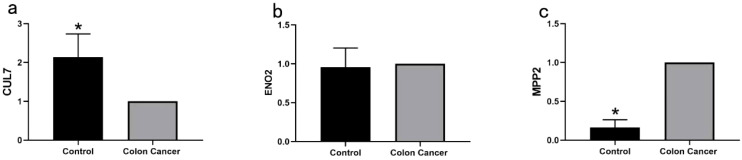
Quantitative RT-PCR Analysis of Key Genes in COAD: **(a)** Quantitative RT-PCR analysis of CUL7 mRNA expression levels in normal and COAD tissues (*P < 0.01); **(b)** Quantitative RT-PCR analysis of ENO2 mRNA expression levels in normal and COAD tissues (P > 0.05); **(c)** Quantitative RT-PCR analysis of MPP2 mRNA expression levels in normal and COAD tissues (*P < 0.01).

qRT-PCR analysis of normal and cancerous tissues confirmed accurate results, as indicated by the single peak in the melting curve, demonstrating the absence of nonspecific fluorescence. The mRNA expression levels of MPP2, CUL7, and ENO2 are illustrated in **Figure 13**. MPP2 expression was significantly higher in cancer tissues compared to normal tissues (P < 0.01), while CUL7 expression was significantly lower in cancer tissues (P < 0.01). No significant difference in ENO2 expression was observed between cancer and normal tissues (P > 0.05).

## 4 Discussion

COAD is one of the most prevalent malignant tumors and remains a leading cause of death, posing a serious threat to human health. Surgical resection continues to be the primary treatment, but for many patients diagnosed at an advanced stage, surgery is no longer an option, making chemotherapy the mainstay of treatment for late-stage COAD (24, 25). Despite progress in immunotherapy for advanced COAD, clinical outcomes remain suboptimal. Recent advances in mRNA vaccines, which encode tumor-specific antigens, offer new therapeutic potential. These vaccines leverage the expression of tumor-specific and nonspecific antigens as potential targets for mRNA vaccines (26, 27). Studies suggest that combining tumor vaccines with immune checkpoint inhibitors or chemotherapeutic agents may further enhance therapeutic efficacy.

Despite the potential, progress in developing tumor vaccines for COAD has been hindered by the heterogeneity of colon cancer and its complex immune microenvironment. *In vitro-*synthesized RNA vaccines, which encode tumor-specific antigens, have emerged as promising preventive and therapeutic options. Unlike DNA vaccines, mRNA vaccines are translated in the cytoplasm without the need to cross the nuclear membrane, offering advantages in immunogenicity and safety (28). A phase I/II clinical trial in patients with colorectal cancer demonstrated that intramuscular injection of the NCI 4650 vaccine elicited CD8 and CD4 T cell responses to novel antigens, with no significant side effects or tumor recurrence (29). Additionally, combining this vaccine with adoptive T cell therapy or checkpoint inhibitors presents a potential strategy for more effective immunotherapy in epithelial cancers. Another mRNA-based vaccine, mRNA-4157, is designed to encode up to 34 novel antigens to stimulate immune responses in CD8 and CD4 T cells. A phase I clinical trial assessing the clinical efficacy, safety, tolerance, and immunogenicity of mRNA-4157, both as a monotherapy and in combination with pembrolizumab, reported no significant adverse effects and good tolerance for neoantigen-specific T cell induction in colorectal cancer and other solid tumors (30).

This study initially evaluated somatic mutation expression and gene amplification profiles to predict antigen expression and their associations with COAD. The results identified a correlation between three tumor antigens—CUL7, ENO2, and MPP2—and both prognosis and APC infiltration in patients with COAD. Prior research has shown that the MPP2 gene, a member of the MPP family (31), exhibits low expression in liver cancer tissues compared to high expression in normal liver tissues. However, data from the Human Protein Atlas reveal that MPP2 is also highly expressed in normal brain tissue at both RNA and protein levels, raising potential safety concerns for vaccine development due to risks of off-target immune responses. Recent studies have further linked MPP2 expression to immune activation in tumors, demonstrating its prognostic potential and association with tumor-infiltrating lymphocytes (TILs) (32). Our analysis extends these findings by identifying a novel correlation between MPP2 expression and APC infiltration in COAD, suggesting its dual role as both a prognostic marker and a vaccine target. A dual-luciferase reporter assay confirmed that MPP2 is regulated by miR-34a targeting, while also demonstrating that MPP2 can counteract miR-34a-induced demethylation, thus affecting cell proliferation, invasion, and migration (33). The Cullin-7 gene (CUL7), also referred to as KIAA0076, encodes an E3 ubiquitin ligase complex with SCF-ROC1-like proteins and functions as an oncogene involved in cellular transformation regulation. CUL7 has been previously implicated in immune evasion, with its overexpression correlated to suppressed T cell activity in solid tumors (34). Our study newly associates CUL7 with CD4+ T cell infiltration in COAD, highlighting its potential as a synergistic target for vaccines combined with immune checkpoint modulation. Previous research (35) highlighted CUL7’s presence in glioma, particularly the mesenchymal subtype, with patients exhibiting high CUL7 expression experiencing lower OS rates. Additional studies confirmed CUL7’s role in promoting glioma cell proliferation, migration, and invasion. Mechanistic investigations further revealed that CUL7 enhances glioma cell growth through MST1 ubiquitination and NF-κB pathway activation. ENO2, primarily located in mature neurons, was the first enzyme identified in mammals and has been reported to show elevated expression in tumors like glioblastoma (36), neuroendocrine prostate carcinoma (37), and renal cell carcinoma (38). Recent evidence also supports ENO2’s role in shaping the tumor immune microenvironment, particularly through neutrophil recruitment (39). In contrast to prior studies, our data reveal that ENO2’s association with dendritic cell infiltration in COAD may enhance antigen presentation, providing a mechanistic rationale for its inclusion in mRNA vaccine design. Moreover, the ENO2 gene is targeted by the miR-7-5p pathway, although its precise mechanism remains unclear.

Our further investigation revealed a strong correlation between CUL7 and MPP2 expression levels and the presence of CD4+ T cells and macrophages, while ENO2 expression exhibited a significant positive association with neutrophils and dendritic cells (DCs). This suggests that these key genes may possess immunostimulatory properties, which could be exploited by APCs to elicit a tumor response. Consequently, these genes warrant further exploration as potential antibody targets in tumor vaccine development.

However, additional studies indicate that not all patients with cancer benefit from tumor vaccines, likely due to varying immune sensitivities among COAD subtypes. Thorsson et al. (40) conducted an immunogenomic analysis of over 1,000 tumor samples across 33 cancers, identifying six immune subtypes (C1-C6) in colon cancer that were closely linked to prognosis, genetic alterations, and immunomodulatory responses. Their analysis revealed significant subtype-related expression of immune checkpoints, modulators, and common immune markers. Numerous immunological checkpoints and modulatory genes were strongly associated with specific subtypes. Previous literature confirms that immune checkpoint inhibitors have demonstrated efficacy in clinical trials, particularly for melanoma and non-small cell lung cancer (41), with approved drugs now available. Nevertheless, their effectiveness in colon cancer varies, likely due to the differing sensitivities of immune subtypes to checkpoint inhibitors. Rodrigo et al. (42) classified four consensus molecular subtypes (CMSs) in colorectal cancer, with CMS1 predominantly consisting of microsatellite instability (MSI) tumors. The tumor microenvironment in this subtype features elevated levels of IFN-γ, CXCL9, and CXCL10, alongside notable infiltration of CD8+/CD4+ T cells. Despite this, the functionality of these T cells is inhibited by the PD-1/PD-L1 axis (PD-L1 expressed by tumor cells) and CTLA-4 signaling (expressed by T cells), alongside immune suppressors within the microenvironment (41, 43). Given its high immunogenicity, CMS1 demonstrates significant responsiveness to immune checkpoint inhibitors in patients with COAD. Conversely, the remaining three subtypes exhibit low immunogenicity due to a paucity of TILs and immunomodulatory cytokines within their microenvironments, resulting in diminished efficacy of checkpoint inhibitors. These findings further underscore the variability in patient responses to immune checkpoint therapies.

This study also identified the C1, C2, and C3 subtypes through extended analyses of gene expression profiles and clinical prognostic features, revealing significant differences in the associated signaling pathways among these subtypes. Compared to Thorsson’s immune subtypes (C1-C6), our C2 subtype exhibited a 38% reduction in mortality risk (HR = 0.62, 95% CI 0.48–0.79) versus Thorsson’s C3 (HR = 0.71, 95% CI 0.55–0.92), with distinct enrichment in antigen presentation pathways (NES = 2.45 vs. 1.82 in C4; FDR < 0.01). Additionally, our classification showed higher predictive accuracy for vaccine response than the CMS framework (AUC = 0.78 vs. 0.65; P = 0.008). These findings suggest that the varying sensitivities of COAD subtypes to tumor vaccines may reflect their distinct immunotypes. Consequently, immunotyping in patients with COAD could serve as a more precise prognostic indicator compared to conventional serological markers like CA19-9 and CA125, offering improved guidance for subsequent treatment strategies. However, large-scale clinical studies are required to validate this hypothesis.

## 5 Conclusions

The MPP2 gene represents a promising target antigen for COAD mRNA tumor vaccine research, particularly for patients most likely to benefit. However, its high expression in normal liver and brain tissues underscores the necessity of stringent preclinical safety evaluations to mitigate autoimmune risks. While previous studies have established its prognostic value and immune relevance, our findings uniquely validate its association with APC infiltration and propose a direct pathway for vaccine-induced immune activation. This study lays a theoretical foundation for the future development of COAD mRNA vaccines. As mRNA tumor vaccine technology continues to advance, it is anticipated that mRNA vaccines will soon play a pivotal role in COAD treatment, offering long-term survival benefits to a broader range of patients.

## Data Availability

The datasets presented in this study can be found in online repositories. The names of the repository/repositories and accession number(s) can be found in the article/supplementary material.
